# Inactivation of RNA and DNA viruses in water by copper and silver ions and their synergistic effect

**DOI:** 10.1016/j.wroa.2020.100077

**Published:** 2020-11-05

**Authors:** Mona Y.M. Soliman, Gertjan Medema, Boris Estrada Bonilla, Stan J.J. Brouns, Doris van Halem

**Affiliations:** a Department of Water Management, Delft University of Technology, Stevinweg 1, 2628, CN Delft, the Netherlands; b KWR Watercycle Research Institute, P.O. Box 1072, 3430 BB, Nieuwegein, the Netherlands; c Department of Bionanoscience, Kavli Institute of Nanoscience, Delft University of Technology, Van der Maasweg 9, 2629, HZ Delft, the Netherlands; d Fagenbank, Van der Maasweg 9, 2629, HZ Delft, the Netherlands

**Keywords:** Metals, MS2 bacteriophage, PhiX 174, Water, Virus, Synergy

## Abstract

Cu and Ag have been used as bactericidal agents since ancient times, yet their antiviral capacity in water remains poorly understood. This study tested the effect of copper (Cu) and silver (Ag) on model RNA and DNA viruses MS2 and PhiX 174 in solution at pH 6–8. Cu caused MS2 inactivation with similar rates at pH 6 and 7 but was inert towards PhiX 174 regardless of pH. Ag inactivated both viruses, causing denaturation of MS2 and loss of capsid spikes in PhiX 174. Ag inactivation rates were pH dependent and increased with increasing pH. At pH 8, 6.5 logs of PhiX were inactivated after 3 h and 3 logs of MS2 after only 10 min. The combined use of Cu and Ag revealed synergy in disinfecting MS2 at pH ≥ 7. Although metal concentrations used were higher than the desired values for drinking water treatment, the results prove a promising potential of Cu and Ag combinations as efficient viricidal agents.

## Highlights

• MS2 (ssRNA) was sensitive to cupric ions, but PhiX 174 (ssDNA) was not.
• Ag^+^ inactivation rate of MS2 and PhiX 174 in water was pH dependent.
• Ag^+^ caused denaturation of MS2 and loss of PhiX 174 capsid spikes.
• At solution pH of 7 and 8, combined use of Cu^2+^ and Ag^+^ had a synergistic effect on MS2 inactivation.

## Graphical abstract

**Figure:**
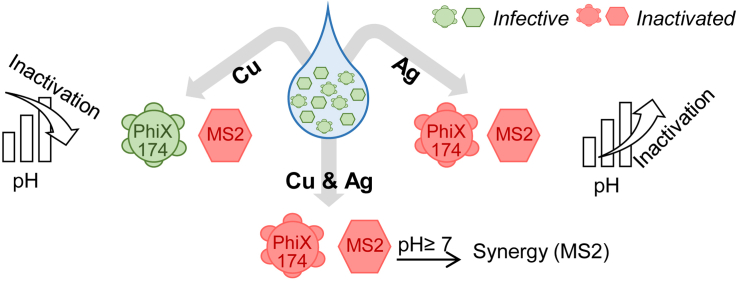

## 1 Introduction

Use of metals in medicine, food and water preservation is dated back to antiquity (Arendsen et al., 2019; Gadi and Jeffrey, 2009; Konieczny and Rdzawski, 2012). Some metals such as copper (Cu) and silver (Ag) display potent biocidal activity against bacteria (Borkow and Gabbay, 2005; Hwang et al., 2007; Pathak and Gopal, 2012; Vincent et al., 2016; Wijnhoven et al., 2009), yeast, fungi (Marambio-Jones and Hoek, 2010) and viruses (Borkow and Gabbay, 2005; Silvestry-Rodriguez et al., 2007). Recent studies have proven the ability of metals to disinfect multidrug-resistant bacteria (Bauer et al., 2019; Kaneko et al., 2007; Mehtar et al., 2008; Mikolay et al., 2010; Wright et al., 1998), control biofilms (Harrison et al., 2004; Lehtola et al., 2004) and have a synergistic effect when used with other biocides (Harrison et al., 2008). This has boosted application of metals for disinfection in a multitude of sectors. In the water sector, Cu–Ag ionization units are used globally to control *Legionella* and other waterborne microbes that may grow in hospital water systems (Borkow and Gabbay, 2005; Stout and Yu, 2003; Triantafyllidou et al., 2016). Cu–Ag ionization was proven more effective than other disinfectants such as UV light and chlorine (Stout and Yu, 2003; Triantafyllidou et al., 2016). The absence of harmful by-products from Cu and Ag has expanded their application to reduce chlorine use in swimming pools (Silvestry-Rodriguez et al., 2007; Yahya et al., 1992). Cu and Ag have also been incorporated in household water treatment systems to improve their microbial disinfection capacity (Oyanedel-Craver and Smith, 2008; Pooi and Ng, 2018).

Although Cu and Ag antiviral activity was first reported in 1963 and 1964 (Yamamoto et al., 1964), research evaluating their effectiveness has been limited. There is a surprising absence of studies evaluating physical and chemical parameters that affect Cu and Ag antiviral activity. Reports evaluating Cu as antiviral agent focused mainly on Cu doses (Armstrong et al., 2017; Nieto-Juarez et al., 2010) and the role of hydrogen peroxide (H_2_O_2_) in enhancing inactivation rates (Kim et al., 2015; Nguyen et al., 2013; Nieto-Juarez et al., 2010; J. L. Sagripanti et al., 1993; Yamamoto, 1969). Cu speciation was briefly addressed by Nieto-Juarez et al. (2010) since inactivation rates of MS2 were associated with the dissolved fraction of Cu ions. Although Ag antiviral studies reported on different doses, comparing results is challenging as different studies used different solution matrices and speciation of Ag^+^ ions was commonly overlooked (Butkus et al., 2004; Kim et al., 2008; Swathy et al., 2014; Zodrow et al., 2009).

The variation in testing conditions was also accompanied by contradicting results on Cu and Ag antiviral efficiency and inactivation capacity. This highlights the need for a systematic evaluation of chemical physical parameters influencing Cu and Ag virucide efficiency as an essential step towards their application. Parameters such as pH, availability of dissolved Ag^+^ and Cu^2+^ ions versus their precipitates has been shown to significantly influence inactivation rates of bacteria (Choi et al., 2009; Lemire et al., 2013; Lin et al., 2002; Lok et al., 2007; Sharan et al., 2010; Xiu et al, 2011, 2012). While similar evaluation of those parameters remains elusive for viruses, a notable effect on virus inactivation can be anticipated.

The speciation of metals is a key element in determining their bioavailability, hence their toxicity and disinfection capacity (Lemire et al., 2013; Workentine et al., 2008). The chemical state of the metal is also subjected to change by environmental factors such as pH, temperature, ionic strength and redox potential (Albrecht et al., 2011; Delahay et al., 1951; Lemire et al., 2013). Changing pH can also change virus conformation and its susceptibility to disinfection, making it important factor in evaluating Cu and Ag antiviral efficiency (Thurman et al., 1989). Moreover, susceptibility to disinfectants differs for different virus types. For example, the single-stranded DNA (ssDNA) phage PhiX 174 was found to be more resistant to heat inactivation than double-stranded DNA (dsDNA) adenovirus (Schijven et al., 2019) and RNA enteroviruses were more resistant to chlorine disinfection than dsDNA adenovirus (Cromeans et al., 2010).

Therefore, the objective of this study was to determine the efficiency of Cu and Ag ions as antiviral agents in water. Conservative model viruses MS2 phage (single-stranded RNA, ssRNA) and PhiX 174 (ssDNA) were selected as targets. Both Cu and Ag were tested individually and in combination in a solution matrix where pH was used as variable. Concentrations of Cu and Ag used were higher than the WHO recommendations to allow for a more feasible evaluation of metals speciation by analytical measurements. The effect of pH on metal ions availability and speciation was evaluated using experimental analysis and the chemical speciation model CHEAQS. Finally, morphology of MS2 and PhiX 174 particles was examined before and after treatment using transmission electron microscopy (TEM) to provide insights on possible structural damage by Cu and Ag.

## 2 Materials and methods

### 2.1 Reagents

All chemicals were reagent grade and used without further purification. Copper sulfate (0.1 M), sodium phosphate monobasic monohydrate (NaH_2_PO_4_.H_2_O), sodium phosphate dibasic heptahydrate (Na_2_HPO_4_.7H_2_O), sodium thioglycolate, Phosphate-Buffered Saline tablets (PBS) were purchased from Sigma-Aldrich. Sodium thiosulfate solution (0.1 M) was purchased from Merck and ethylenediaminetetraacetic acid solution (EDTA) 0.1 M and silver nitrate (0.01 M) from VWR.

All experimental solutions were prepared using Milli-Q water (18 MΩ, pure lab chorus 1). Sodium phosphate buffer (PB, 1 mM) contained NaH_2_PO_4_.H_2_O and Na_2_HPO_4_.7H_2_O mixed at concentrations of 0.93 mM and 0.07 mM for pH 6, 0.58 mM and 0.42 mM for pH 7 and 0.12 mM and 0.88 mM for pH 8, respectively. Solutions were autoclaved at 121 °C for 20 min then stored at 4 °C until further use. When needed, further adjustment of pH was conducted using stock solution NaH_2_PO_4_.H_2_O (100 mM) as a base and Na_2_HPO_4_.7H_2_O (100 mM) as an acid. Silver neutralizing solution was prepared on the experimental day as described by Butkus et al. (2004). The solution contained 12 g/l of sodium thioglycolate and 0.1 M/l of sodium thiosulfate.

### 2.2 MS2 and PhiX 174 phage culturing and purification

MS2 stock was produced by infecting *Escherichia coli* C3000 (ATCC 15597), grown to early logarithmic phase (OD_600_ 0.1–0.2), with MS2 suspension (ATCC 15597-B) at multiplicity of infection (MOI) 0.01 phage per cell. Following overnight replication (37 °C; 30 rpm), the lysate was harvested by centrifugation (Sorvall™ ST 16 R, 4000×*g*, 20 min, 4 °C). The aspirated supernatant was filter sterilized through 0.22 μm sterile syringe filters (PES, VWR) then stored at 4 °C. Similarly, *E. coli* WG5 (DSM 18455) was infected at 0.2 OD_600_ by PhiX 174 suspension (DSM 4497) at MOI 0.01 and incubated overnight at 37 °C and 110 rpm for replication. Lysate was harvested and processed as described for MS2.

Both phage stocks were further purified by density gradient ultracentrifugation to eliminate bacterial protein residuals, broth organics or any impurities that can intervene with evaluating metals inactivation capacity. Moreover, density gradient purification reduces phage aggregation (Dika et al., 2013) which (if occurred) affects inactivation rates (Gerba and Betancourt, 2017). MS2 was suspended in iodixanol gradient (OptiPrep™- Stemcell) and PhiX 174 in cesium chloride; each adjusted to 20% (w/v) underlined by 40% and 60% of corresponding solution. The gradient was centrifuged for 20 h (32,000 rpm, 4 °C, Beckman coulter optima L-90 K ultracentrifuge). The corresponding phage band was gently removed using syringe needle then re-suspended in PB buffer (pH 7). The suspension was washed twice using 30 kDa and 100 kDa Amicon® Ultra columns; centrifuged 4000×*g*, 20 min (Wick and McCubbin, 1999). Recovered phage was stored in PB buffer (pH 7) at 4 °C until further use. Enumeration of both phages, agars and cultures used were done according to the ISO- 10705 part 1 and 2. Final concentration of MS2 and PhiX 174 produced suspension were 10^13^ pfu/ml and 10^10^ pfu/ml respectively.

### 2.3 Inactivation experiment

Through the inactivation experiment, interaction between metals and MS2 or PhiX 174 was examined in pH and temperature controlled (25 °C) inorganic buffer (PB). The reaction took place in sterilized acid washed (10% nitric acid) glass beakers. Beakers were wrapped in aluminium foil and covered from the top with a sterile petri-dish and foil to ensure dark conditions.

MS2 and PhiX 174 were tested separately each for inactivation by Cu, Ag, or both Cu and Ag at pH 6, 7 and 8. Additionally, interaction between MS2 and Ag was tested at pH 7.5. All conditions were examined at least in triplicates. In total 26 metal inactivation experiments were conducted of which 7 were metal free control tests.

On the experimental day, test water was prepared by adding phage suspension to PB buffer (pH 6, 7 or 8) to a final concentration of approximately 10^6^ pfu/ml and a 100 ml of test water were added to each beaker. For metal testing, the reaction was initiated by adding aliquots of metals to each beaker for a final concentration of Ag 4.6 μM (0.5 mg/l) and 78.7 μM (5 mg/l) for Cu. Solutions were placed inside a 25 °C incubator and stirred continuously at 60 rpm (2mag-Magnetic-Drive).

At time intervals 0, 0.17, 0.5, 1, 3 and 6 h, a 5 ml of sample were withdrawn using sterile syringes and directly neutralized to stop the reaction. Frequency of sampling was increased in some experiments. Ag containing samples were neutralized using 2 mM of silver neutralizer (Butkus et al., 2004), Cu containing samples were neutralized with 5 mM of EDTA, and Cu and Ag samples were neutralized with both solutions. Metal-free control samples were also neutralized to examine the neutralizer effect on phage recovery. After neutralizing, samples were stored on melting ice and MS2 samples were diluted in PBS buffer, PhiX 174 in PB buffer. Phage enumeration followed the double agar layer method (DAL) by assaying duplicates of 1 ml sample and serial sample dilutions as described in ISO-10705. Limit of detection (LOD) was 1 pfu/ml and the lowest concentration (LOQ) considered reliable was 30 pfu/ml as recommended by the ISO-10705. Reported results represent the average values and standard deviations of triplicate tests. In addition, samples from metal beakers were analysed to confirm metals concentration using 1CP-OES Spectrometer (Spectro Arcos eop).

### 2.4 Data analysis

Log removal values were calculated as Log_10_ (N_t_/N_0_) where N_t_ is the phage concentration (pfu/ml) at time t and N_0_ is the concentration at time 0. First order inactivation rate constant, K_obs_ (h^−1^) was calculated from the slope of linear regression of Ln (N_t_/N_0_) versus time (h). Linear regression analysis was used to calculate the Kobs with 95% confidence interval and determine the significance of slope variation from zero. Linear regression slopes of metal free controls were compared to zero for significance of any noticeable decay. K_obs_ (mean value, SD and df) for different conditions was compared using one-way ANOVA with Tukey.

### 2.5 Speciation of metal ions

The ICP-OES provides a measurement of the total metal ions without distinction between ionic, dissolved or solid precipitates. The speciation of metals is important since toxicity is often related to the metal’s ionic form rather than dissolved or solid complexes (Pham et al., 2012). Thus, a filtration test first provided an experimental assessment of dissolved metals vs solid complexes through solids exclusion by retention on the filter. Metal samples were filtered using a series of syringe filters (0.45 μM, 0.22 μM, 0.1 μM and 0.02 μM; Polyethersulfone PES), sampled after passing each filter and analysed using ICP-OES. The same was repeated on metal samples diluted in milliQ which was used as control to evaluate possible metal adsorption to the filters.

Concentration of the unfiltered samples: Ag 4.6 μM (0.5 mg/l) and 78.7 μM (5 mg/l) for Cu was used as the total concentration. Lowest filtrate concentrations were considered dissolved and the difference between the total and dissolved is the filtered solid complexes. Average values and standard deviation are reported. All values are reported as a percentage (%) of the total metal concentration added.

The CHEAQS Next chemical equilibrium program was used to calculate the free ionic concentrations (Ag^+^ and Cu^2+^) in the buffer matrixes used. The measured total concentrations of Cu, Ag, Na and PO_4_ were used as input together with measured pH and redox potential (SenTix ORP-T 900).

### 2.6 TEM imaging

The morphology of phage particles was examined using transmission electron microscopy (TEM, JEOL JEM-1400 plus). Since high phage concentration is required for TEM, a sub experiment was conducted using phage suspensions at 10^9^ and 10^11^ pfu/ml for PhiX 174 and MS2, respectively. In brief, 100 μl of phage suspension were treated with either Ag or Cu or both. The concentrations used were approximately 20 μM of Ag and 3 mM Cu. The suspension was incubated as mentioned above but in 2-ml microcentrifuge tubes. Prior to TEM grid preparation, PhiX 174 sample buffer was exchanged for milliQ by centrifugation (21,000×*g*, 50 min, 4 °C). A second centrifugation step was applied to further wash the sample and the supernatant was discarded. For MS2 and PhiX 174, a 10 μl of the sample was incubated for 5 min on a copper mesh (Carbon Type-B, 400 mesh, TED PELLA). The excess liquid was extracted using filter paper, and the sample was stained using 2% uranyl acetate for 30 s. Imaging was repeated twice for each sample to ensure reproducibility, additional information and images are provided in supplementary information.

## 3 Results

### 3.1 Effect of pH on metal free controls

**Fig. 1:**
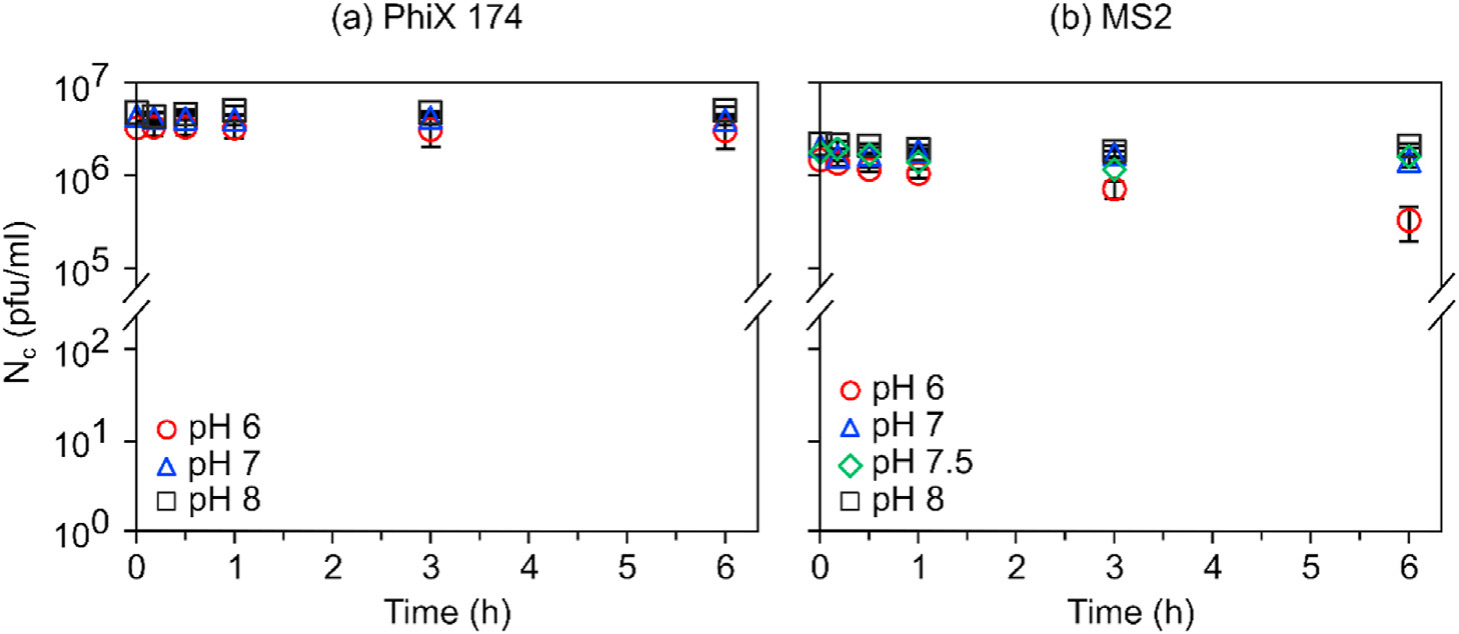
Stability of (a) PhiX 174 and (b) MS2 phage in metal free PB buffer at pH (6–8) and 25 °C.

To evaluate the stability of PhiX 174 (ssDNA) and MS2 (ssRNA) under experimental conditions, metal free controls were sampled and analysed as metal samples. Concentrations of PhiX 174 (Fig. 1a) were stable during the 6 h testing period at each tested pH; K_obs_ similar (p > 0.05) to slope zero. Same was observed for MS2 at pH ≥ 7. At pH 6, MS2 metal free control decayed over 6 h with K_obs_ = 0.22 ± 0.01 (h^−1^) significantly different from zero slope (p < 0.0001). To properly evaluate the inactivation caused by metals at pH 6, MS2 inactivation results were corrected to account for the observed decay in the control experiments.

The correction was applied by calculating log removal values (LRV) as Log_10_ (N_t_/N_c_) where N_t_ is the phage concentration of test condition at time t and N_c_ is the phage concentration of control condition also at time t. K_obs_ was calculated as the slope of Ln (N_t_/N_c_).

### 3.2 Metal ions complexation

The concentration of dissolved Cu^2+^ and Ag^+^ was measured and compared against the modelling results of *CHEAQS next* chemical software. The concentration of ionic, dissolved, or solid metal ions is expressed as a percentage of the total experimental concentration; 4.6 μM (0.5 mg/l) for Ag and 78.7 μM (5 mg/l) for Cu. This provides more insight on ions availability (free ionic form) and complexation which might reflect on the antiviral activity of the metals.

**Fig. 2:**
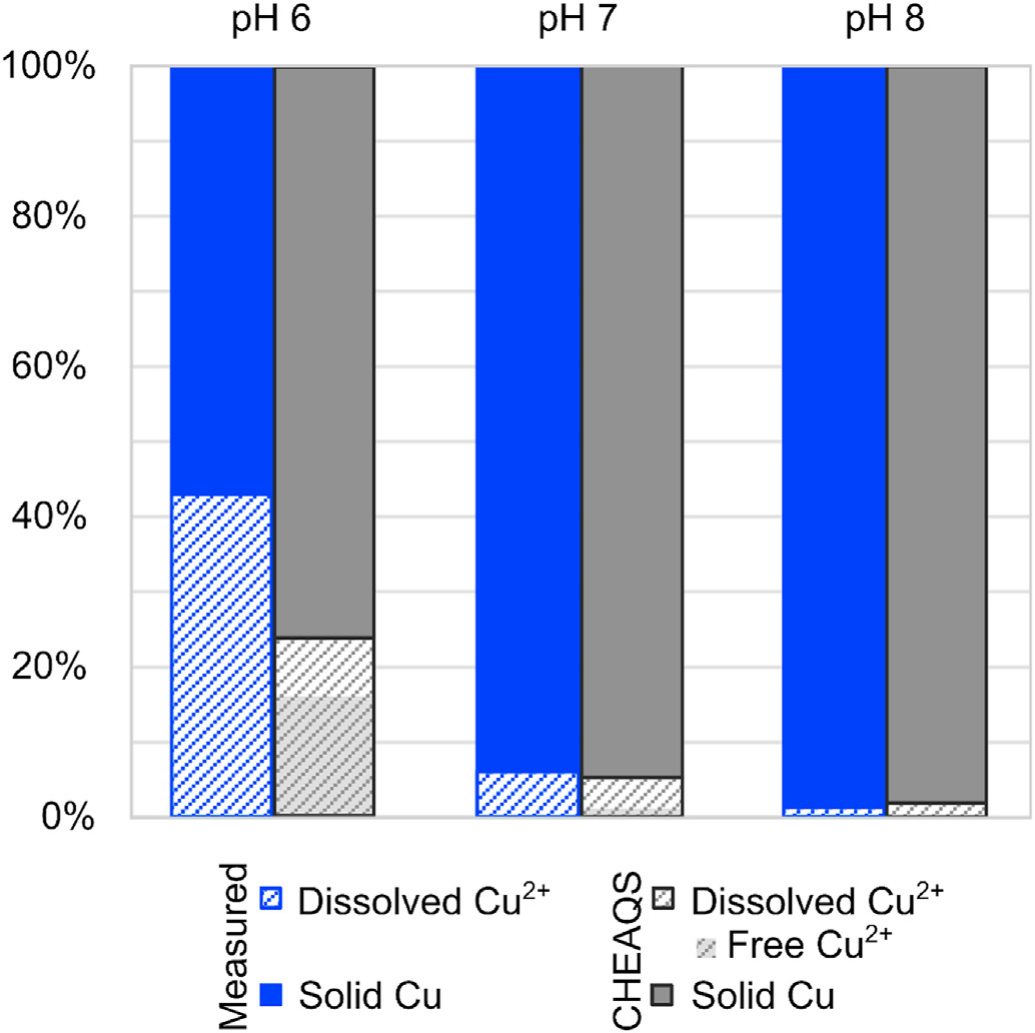
Speciation of Cu ions in solution expressed through experimental measurements of dissolved and solid Cu concentrations referred to as (measured), compared to the model speciation output using CHEAQS next chemical software referred to as (CHEAQS). Cu measured concentrations after 0.02 μm filtration are expressed as dissolved, while the difference between dissolved and total concentration (unfiltered sample) is considered solid Cu. Values are expressed as a percentage of the total Cu concentration which was used also as the input for CHEAQS.

Changes in solution pH had a clear effect on speciation of Cu and availability of Cu^2+^ ions. CHEAQS modelling results show a change in dissolved Cu from 24% at pH 6, to 5% at pH 7 and 2% at pH 8, of which 16, 1 and 0.2% are free ionic Cu^2+^ respectively (Fig. 2). Experimental measurement of dissolved Cu yielded different percentages of dissolved Cu but the trend did align with the model predictions. Measured dissolved Cu at pH 6, 7 and 8 were 43%, 6% and 1% respectively. The higher percentage of experimentally measured dissolved Cu^2+^ could be due to the formation of copper particles smaller than the filter pore size (<20 nm), which are capable of passing the filter and being analysed as dissolved. Yet, both methods (model and experiment) confirm a higher percentage of available dissolved Cu^2+^ at lower pH.

For Ag, changing pH had no effect on Ag^+^ speciation (supplementary Figure S1). CHEAQS predictions show 100% dissolved Ag at all pH values, of which free ionic Ag^+^ changed from 100% at pH 6–t99% at pH 7 and 97% at pH 8. This slight drop was due to the formation of AgH(PO_4_)^-^ (Figure S1). Analytical measurements also showed 100% dissolved Ag (Figure S1). The results for Cu and Ag in the combined solutions were identical to those of Cu and Ag alone.

### 3.3 Viral inactivation by Cu

**Fig. 3:**
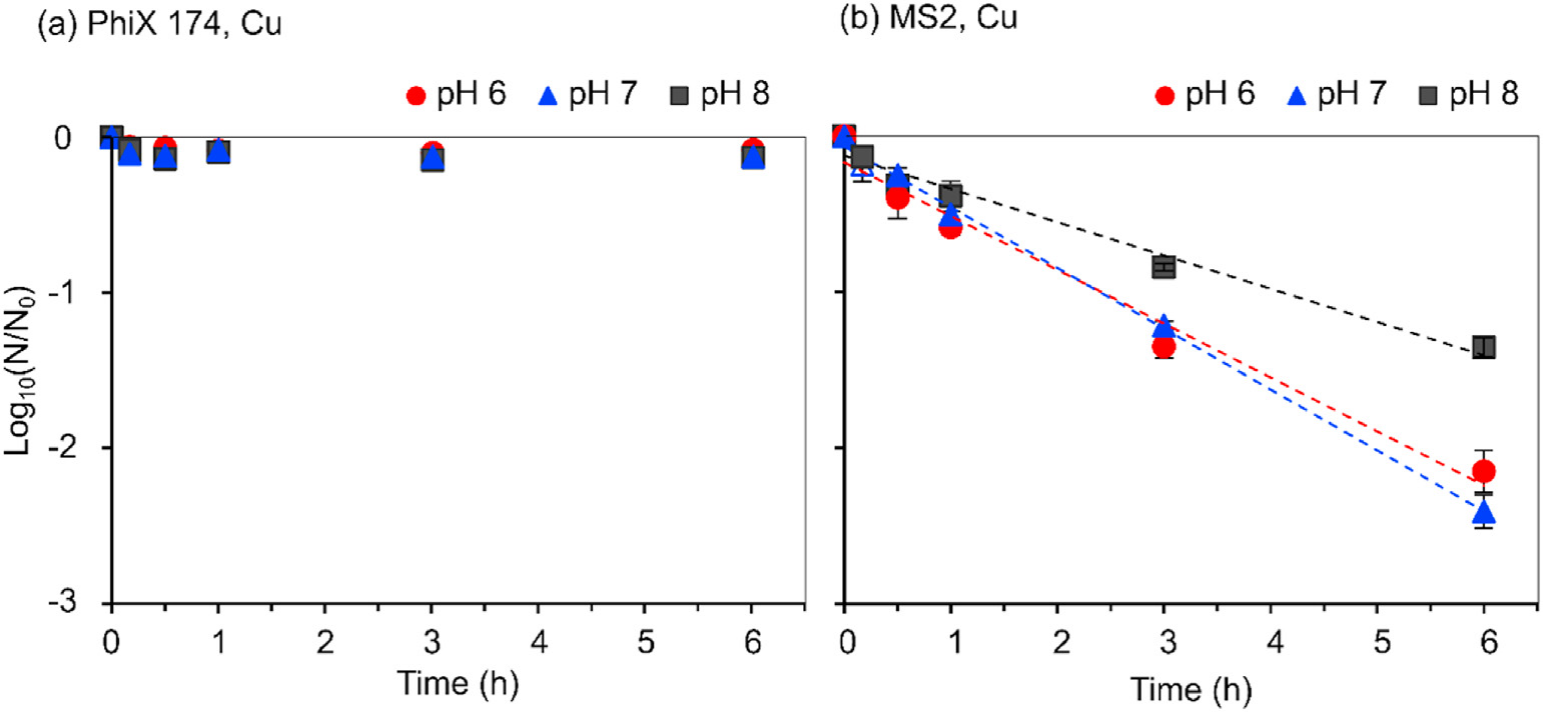
Copper inactivation of (a) PhiX 174 and (b) MS2 phage at pH 6–8. Inactivation rate constants: Kobs (h^−1^) ± standard deviation (SD) for MS2 are mentioned in the text. R^2^ values were >0.97 for all Kobs values. Regression lines are shown in same colour as the pH markers. (For colour interpretation, the reader is referred to the web version of the article). (For interpretation of the references to colour in this figure legend, the reader is referred to the Web version of this article.)

Antiviral efficiency of Cu against PhiX 174 and MS2 was compared under acidic (pH 6), neutral (pH 7) and alkaline (pH 8) conditions. Cu treatment caused no inactivation of PhiX 174, regardless of the pH (K_obs_ insignificant from control and from 0 slope, p > 0.05) (Fig. 3a). Inactivation kinetics of MS2 followed first order, Chick Watson model (Fig. 3b). Slowest kinetics were observed at pH 8 (K_obs_ = −0.89 ± 0.004 h^−1^; 1.4 LRV over 6 h); significantly different from K_obs_ at pH 6 and 7 (p < 0.0001). However, statistically similar (p = 0.29) inactivation kinetics at pH 6 (K_obs_ = −0.79 ± 0.06 h^−1^; 2.2 LRV over 6 h) and pH 7 (K_obs_ = −0.90 ± 0.02 h^−1^; 2.4 LRV over 6 h) were observed. K_obs_ values were significantly different (p < 0.0001) from metal free control slopes and zero slope validating that efficiency of Cu in inactivating MS2.

### 3.4 Viral inactivation by Ag

**Fig. 4:**
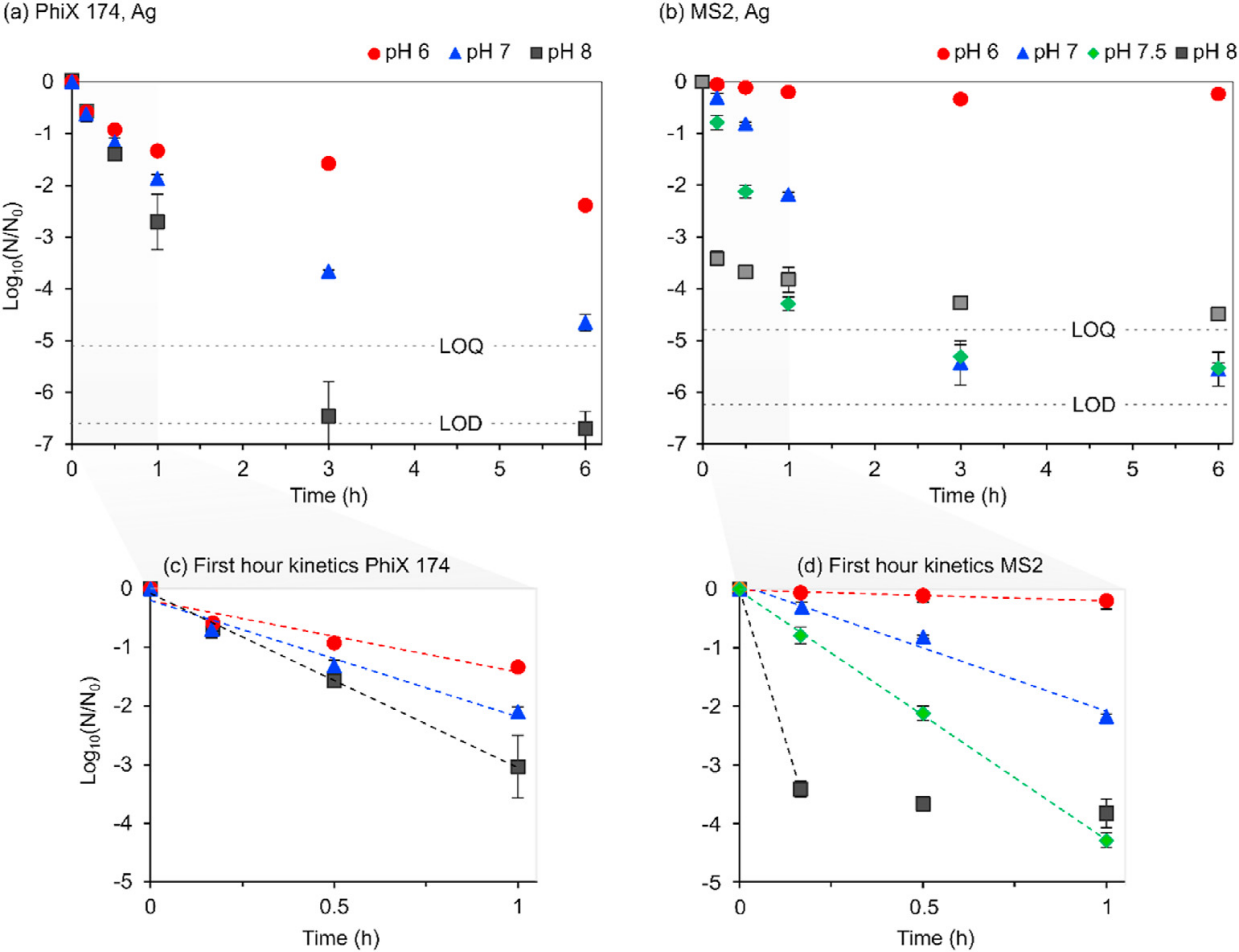
Ag inactivation of PhiX 174 and MS2. Full 6 h LRVs are shown in top row (a and b) while modelling of first hour inactivation kinetics is shown in bottom row (c and d). Limit of detection (LOD) was 1 pfu/ml and the lowest concentration (LOQ) considered reliable was 30 pfu/ml. Kobs (h^−1^) values are mentioned in the text. R^2^ for all Kobs values was >0.96 except for PhiX 174 at pH 6 (R^2^ = 0.90). Regression lines are in same colour as the pH markers (For colour interpretation, the reader is referred to the web version of the article). (For interpretation of the references to colour in this figure legend, the reader is referred to the Web version of this article.)

Ag showed a pronounced antiviral activity on both MS2 and PhiX 174. After 3 h of exposure to Ag ions, PhiX 174 reached the lowest concentration (LOQ) equivalent to 6.5 ± 0.7 logs (Fig. 4a, pH 8). Ag inactivation of MS2 also reached LOQ (∼5.5 ± 0.2 logs) at pH 7 and 7.5 over 3 h (Fig. 4b). To avoid values at LOQ, only the first hour was considered in evaluating inactivation kinetics (Fig. 4c and d).

The slowest inactivation kinetics of MS2 was observed at pH 6 (K_obs_ = −0.43 ± 0.04 h^−1^; ∼0.3 LRV over 1h). K_obs_ of Ag treated MS2 and metal free control at pH 6 were similar (p = 0.94), hence Ag was ineffective against MS2 at pH 6. Meanwhile, significantly higher (p < 0.05) kinetics were observed for PhiX 174 at pH 6, K_obs_ (−2.81 ± 0.65 h^−1^; ∼1.3 LRV over 1h).

At pH 7, inactivation rate constants were similar (p = 0.69) for MS2 (K_obs_ = −4.99 ± 0.48) and PhiX 174 (K_obs_ = -4.09 ± 0.57). The difference in K_obs_ between pH 6 and 7 corresponds to an increase by a factor of 1.5 for PhiX 174 and by a factor 10 for MS2. K_obs_ for PhiX 174 continued to increase with the same magnitude (1.5 times) between pH 7 and 8, reaching −6.3 ± 0.37 h^−1^ at pH 8. Also, K_obs_ of MS2 doubled between pH 7 and 7.5.

Overall, increased K_obs_ by increasing pH was significant (p < 0.0001) and clear pH dependency of MS2 and PhiX 174 inactivation rates was observed. Inactivation kinetics followed Chick-Watson model (Fig. 4c and d), except for MS2 at pH 8. The initial 10 min of interaction between MS2 and Ag resulted in 3.5 LRV followed by a distinct tailing and much slower kinetics (Fig. 4d).

### 3.5 Antiviral activity of Cu and Ag combined

**Fig. 5:**
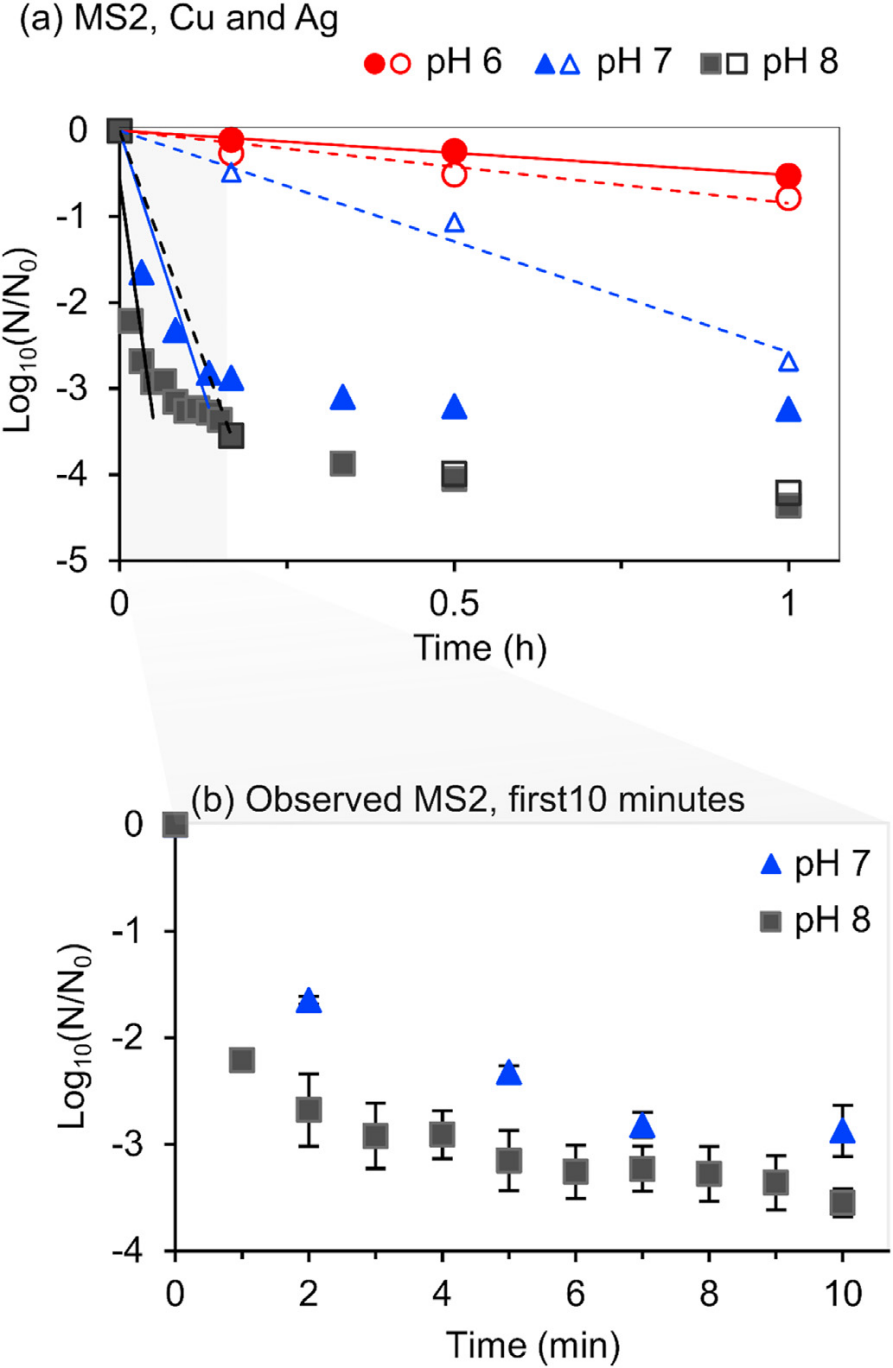
First hour inactivation of (a) MS2 by Cu and Ag ions combined as observed (closed marker) and the mathematical sum of LRVs obtained through individual treatment as estimated (open marker). (b) The first 10 min of observed MS2 inactivation by Cu and Ag. Regression lines are in same colour as the pH markers (For colour interpretation, the reader is referred to the web version of the article). (For interpretation of the references to colour in this figure legend, the reader is referred to the Web version of this article.)

The combination of Cu and Ag was tested to evaluate possible synergies. The experimentally observed LRVs of the Cu and Ag combination are compared to the mathematical sum of LRVs obtained from individual metal treatment, reported as (*estimated*). The first hour results of MS2 are depicted in Fig. 5. Results of PhiX 174 and the 6-h experiment are provided in supplementary material (Figure S2 and S3).

For PhiX 174, observed and *estimated* K_obs_ were similar (p > 0.7) (Figure S3). Also, observed and *estimated* K_obs_ for MS2 at pH 6 were similar (p > 0.9) (Fig. 5a). MS2 observed inactivation kinetics at pH 7 (K_obs_ = −35.95 ± 9.4 h^−1^; ∼2.7 LRV in 7 min) was significantly higher (p < 0.05) than the *estimated* inactivation kinetics (K_obs_ = −5.89 ± 0.84 h^−1^; ∼2.7 LRV over 1h). Similarly, observed K_obs_ was higher at pH 8 than the *estimated* rate constant (47.5 versus 16 h^−1^). This highlights a distinct synergetic effect of using Cu and Ag combined at pH ≥ 7 compared to their individual use.

However, for the two conditions (pH 7 and 8), inactivation of MS2 diverted from first order kinetics following initial drastic rapid inactivation. Observed inactivation of MS2 reached 3 logs after 10 min at pH 7 and 3 logs after 3 min at pH 8, both followed by subsequent tailing that decelerated the inactivation rates (Fig. 5b). MS2 diversion from first order kinetics (tailing) was initially observed using Ag at pH 8 (Fig. 4) which was reflected in the *estimated* LRVs of MS2 at pH 8 (Fig. 5a). In the three experimental settings where MS2 inactivation rapidly reached 3 Logs (in ≤10 min), subsequent tailing was observed.

### 3.6 Morphological changes of MS2 and PhiX 174

TEM imaging was used to examine morphological changes to the phage after metal treatment compared to the intact particles in metal free buffer. The examination aimed to capture possible structural damage resulting from phage interaction with metals.

**Fig. 6:**
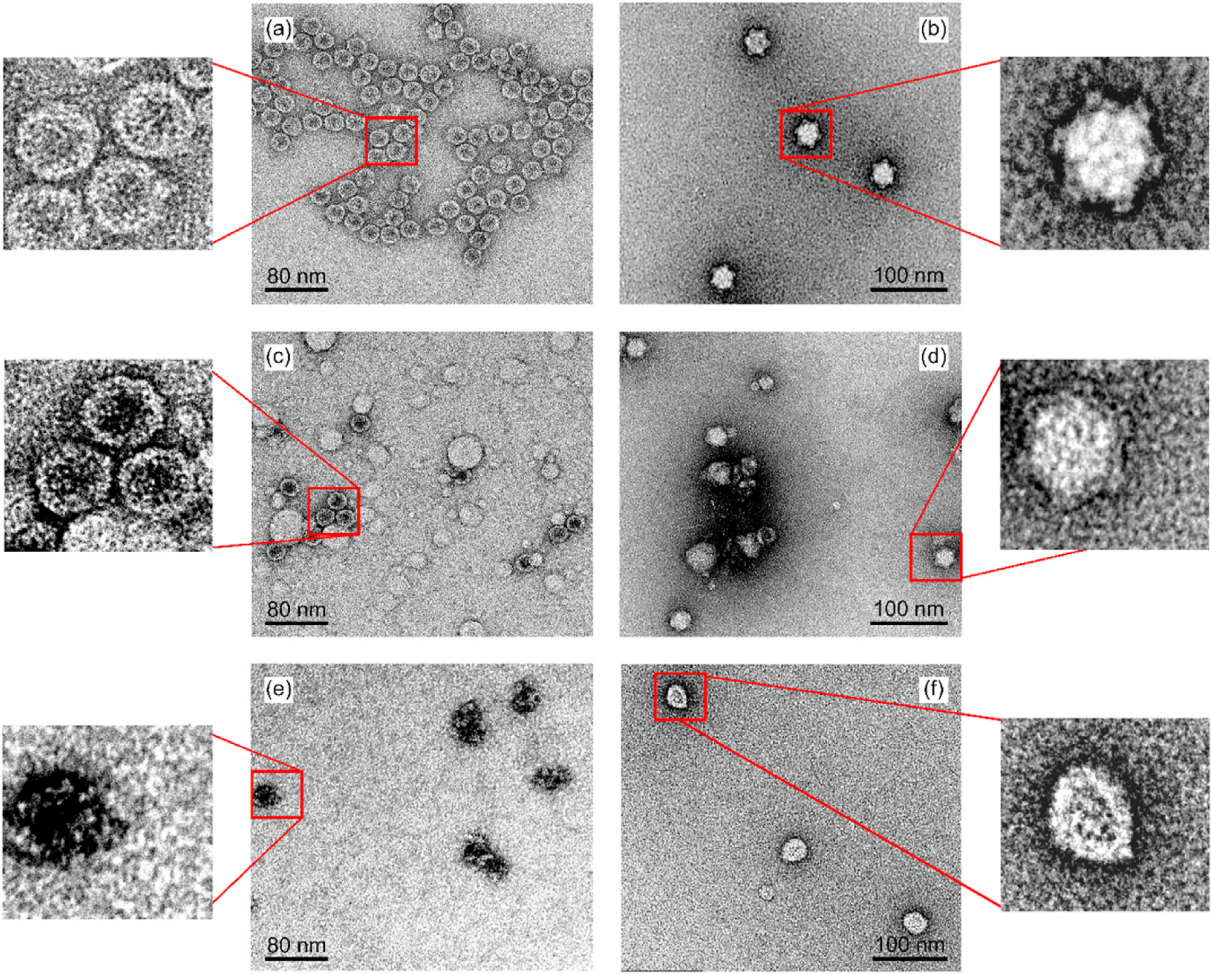
TEM images of MS2 phage (left column) and PhiX 174 (right column). Top row shows control particles (a) MS2 intact circular shape and (b) PhiX 174 intact round shell with spikes. Middle row: Cu treated (c) MS2 with dark capsid centre and white chemical precipitates on the grid and (d) PhiX 174 similar to the control except for presence of chemical precipitates and debris on the grid. Bottom row: Ag treated (e) MS2 with noticeable structural damage as capsid integrity is compromised and the particles denatured and (f) PhiX 174 lost its capsid spikes. MS2 particles were suspended in PB buffer pH 7 and PhiX 174 in PB buffer pH 8. Imaging was repeated twice to ensure reproducibility.

As shown in Fig. 6a, MS2 control particles can be seen with intact round shaped capsid. The PhiX 174 control particles showed clear distinct capsid spikes (Fig. 6b). After exposure to Cu ions, MS2 particles appear with the same round morphology as the control but with darkened centre (Fig. 6c). This darkness is due to stain (uranyl acetate) penetration into the capsid. This could be a result of defective capsid (Swathy et al., 2014) or RNA free capsid (Hooker et al., 2004). The white, non-uniform particles appearing in the image were judged as chemical precipitates appearing on the grid. The Cu treated PhiX 174 (Fig. 6d) showed no difference from the control image except for debris or possible chemical precipitation similar to those in the MS2 image.

The Ag treated MS2 particles, however, showed a clear difference in morphology (Fig. 6e). MS2 particles looked irregularly shaped, losing their round intact structure and looking severely damaged. PhiX 174 particles also appeared distinctly different after Ag treatment (Fig. 6f). Although the centre of the PhiX 174 capsid can still be recognized, a clear loss of capsid spikes and change in particle size was observed.

Finally, no distinguishable additional structural damage was observed in MS2 samples containing both Cu and Ag but rather a combination of non-uniform white precipitates and denatured particles as observed in the Ag image (Image not shown).

## 4 Discussion

### 4.1 Cu inactivation of PhiX 174 and MS2

Cu inactivation of PhiX 174 and MS2 was tested to investigate the potential of Cu ions as an antiviral agent against human enteric viruses in water. Using variable pH (6–8), speciation of Cu was evaluated against inactivation rates achieved. The increase in pH was associated with reduction in free ionic Cu^2+^ and dissolved Cu.

Regardless of Cu^2+^ availability, PhiX 174 was not affected by Cu treatment. No inactivation was observed experimentally, or change in PhiX 174 morphology in TEM images. Sagripanti et al. (1993) also reported no inactivation of PhiX 174 by Cu treatment (15 mM). However, Li and Dennehy (2011) reported 3.5 LRV of PhiX 174 after adding 5 mM of CuSO_4_ to the PhiX 174 lysate. This difference cannot be explained by Cu dose since the higher dose used by Sagripanti et al. (15 mM) and the lower dose (78.7 μM) used in our study did not cause PhiX 174 inactivation.

Of note, Li and Dennehy added Cu to the lysate which contains organics while in our study inorganic buffer was used. The interaction between organics and Cu^2+^ can lead to Cu reduction to more toxic form Cu^+1^ and possible production of H_2_O_2_ (Pham et al., 2012). H_2_O_2_ interacts with dissolved oxygen producing ROS, which causes phiX 174 inactivation by DNA degradation (Lee et al., 2016; Ueda et al., 1980). Cu^2+^ alone reportedly does not cause damage to the genomic DNA unless ROS were produced (Łȩ;czkowska and Vilar, 2013; Lemire et al., 2013; Sagripanti et al., 1991). So, the inactivation effect of Cu on PhiX 174 reported in some studies is most probably caused by a more toxic form of Cu or production of ROS, and Cu^2+^ alone does not cause inactivation of PhiX 174 regardless of its concentration and solution pH.

On the other hand, MS2 was inactivated by Cu at variable rates depending on the pH. Although Cu concentration used in this study was higher than WHO recommendation of 2 mg/l (WHO, 2017), LRVs achieved at same pH might not vary when WHO limit is applied since the reported MS2 inactivation rates at Cu dose ≥1 mg/l were similar due to solution saturation with copper (Armstrong et al., 2017; Nieto-Juarez et al., 2010). According to Nieto-Juarez et al. (2010) inactivation of MS2 by Cu is caused only by its dissolved fraction. Our analysis show that dissolved Cu was higher at pH 6 than at pH 7 by 19% (CHEAQS data). However, the observed inactivation rates of MS2 were similar at pH 6 and 7 and lower at pH 8. In this situation, the effect of pH on MS2 inactivation rates cannot be explained by Cu^2+^ solubility alone.

Potentially, the presence of MS2 in solution can influence the speciation of Cu^2+^. A study by (Badetti et al.2019) found that amino acids can solubilize Cu^2+^ from Cu nanoparticles to form soluble complexes with Cu^2+^. However, the higher inactivation kinetics at pH 7 compared to pH 8 does not support this mechanism either, and point more towards conformational or structural changes in the MS2 capsid.

It has been reported that at pH 6 to 8, most of the Cu^2+^ binds to Histidine (His) amino acid in proteins (Barber-Zucker et al., 2017), and a small fraction to Cysteine (Cys) amino acids (Minoshima et al., 2016). According to Barber-Zucker et al. (2017) increasing pH from 6 to 7 and 7 to 8 enhanced Cu^2+^ interaction with His by 20% and 1% but reduced Cu^2+^ interaction with Cysteine (Cys) amino acid by 2% and 1% respectively. So, it is likely that while pH 6 favoured Cu^2+^ dissolution, pH 7 favoured the interaction between Cu^2+^ and His, resulting in a similar inactivation rates as observed in our study. The lower rates at pH 8 can be attributable to further loss of available Cu^2+^ which is not compensated by an increase of Cu^2+^ interaction with His.

The images of MS2 Cu treated particles showed preservation of the circular capsid morphology with a dark centre, which could be an artificial effect from the Uranyl salts used as negative stain (Wan et al., 2000) or a result of defective capsid protein (Swathy et al., 2014). If Cu caused only conformational change (such as coordinating with amino acids), it would not be visible under TEM, making the artificial interpretation more likely. However, the exact structure damage cannot be confirmed based on the images only so other possibilities cannot be excluded.

### 4.2 Ag inactivation of MS2 and PhiX 174

The results show that Ag is a potent antiviral agent against both MS2 and PhiX 174. Ag inactivation rates followed first order -Chick Watson-kinetics except for MS2 at pH 8 where tailing was observed. Viruses tailing has been commonly explained by aggregation of virus particles induced by solution pH (close to virus isoelectric point), virus type, salt concentrations, bacterial debris, organic matter, type or concentration of disinfectant (Gerba and Betancourt, 2017). The purification of MS2 stock -as described in section 2.2- should remove bacterial debris, organics and significantly reduce aggregation (Dika et al., 2013). Since the same MS2 stock, inorganic buffer (PB, 1 mM) and Ag concentration were used in experiments where tailing was not observed, it is unlikely that aggregation was the cause of observed tailing. While the exact reason behind observed tailing remains unknown, it is not determinative in evaluating Ag antiviral efficiency since it is confined to only one condition (MS2, pH 8).

Inactivation rates by Ag were pH dependant for MS2 and PhiX 174, as K_obs_ increased with increasing pH. Since Ag was available in its ionic free form (Ag^+^) regardless of pH, the observed dependency cannot be explained by Ag speciation. Similar pH dependency of inactivation kinetics of bacteria by Ag was reportedly associated with the increase in the negatively charged sites on the bacteria’s membrane which enhanced the interaction with Ag^+^ ions (Brown and Anderson, 1968; Erickson et al., 1998; Lin et al., 2002; Pathak and Gopal, 2012). In viruses, protonation of amino acids (loss of H^+^) is responsible for the presence of negative sites in the protein capsid depending on its pKa value. Since pKa values differ per protein structure and the solution pH (Poole, 2015), pKa values of MS2 and PhiX 174 amino acids was predicted using PROPKA 3.0 and provided in supplementary (Table S2, S3).

The higher inactivation rates observed at pH 6 for PhiX 174 compared to MS2 can be attributed to the presence of Cys residue with lower pKa value in PhiX 14 spike protein (G) compared to the pKa of Cys in MS2 capsid protein (See supplementary Table S2, S3). Although other amino acids had lower pKa, denaturation of cysteine has been linked to Ag inactivation of viruses in literature (Clem Gruen, 1975; Kim et al., 2008; Minoshima et al., 2016; Xiu et al., 2011; Zodrow et al., 2009). TEM images of Ag treated PhiX showed a loss of the capsid spikes so we only accounted for pKa values of Cys in PhiX 174 spike protein G and Cys pKa in MS2 capsid protein.

The gradual increase of PhiX 174 inactivation rates with increasing pH is in agreement with the literature reported increase of the thiolates/thiols ratio which enhances the interaction between Ag^+^ and Cys (Clem Gruen, 1975; Poole, 2015). Meanwhile, the sharp increase in MS2 K_obs_ at pH ≥ 7 suggests involvement of both Cys and RNA in Ag^+^ mediated inactivation mechanisms. Ag treated MS2 particles observed under TEM support this hypothesis since the capsid is completely distorted. Finally, similar to Cu, the concentration of Ag used in this study was higher than WHO recommendations for drinking water (0.1 mg/l), so lower LRVs can be anticipated when applying Ag in agreement with the WHO guidelines (Butkus et al., 2004).

### 4.3 Inactivation of MS2 and PhiX 174 by Cu and Ag combined

No synergy was observed between Cu and Ag for PhiX 174 inactivation. The combination of Cu and Ag ions exhibited a synergistic effect on MS2 inactivation at pH ≥ 7, but not at pH 6. Ag was only effective in disinfecting MS2 at pH ≥ 7 while Cu has similar efficiency at pH 6 and 7. So, it is likely that synergy is led by Ag ions rather than Cu and for synergy to occur both metals need to be individually effective in virus disinfection. Ag^+^ ions are strongly polarized compared to Cu^2+^, so when both ions are present in solution it is highly likely that Ag^+^ would interact first (Lemire et al., 2013).

Synergy of Cu and Ag in inactivating MS2 was also reported by Yahya et al. (1992) at pH 8, yet the reason behind it remains unclear. Chemical speciation of Cu and Ag was similar to the speciation of individual solutions. Also, TEM images provide no possible explanation for the synergy observed.

Further research is needed to understand the reason behind Cu and Ag synergy. Moreover, mechanistic understanding of the tailing kinetics can help evaluate if virus inactivation by metals could be self-limiting under certain conditions. Although a promising potential of Cu and Ag antiviral efficiency is evident in this study, evaluation of complexing factors such as natural organic matter is an important step for the complete picture of metal’s antiviral efficiency and their potential application in drinking water treatment.

## 5 Conclusions

• Our study has clarified essential aspects of evaluating antiviral activity of metals. Changing solution pH did not only affect the speciation of Cu but also the sensitivity of viruses to disinfection.
• Free ionic Ag^+^ strongly inactivated ssRNA MS2 and ssDNA PhiX 174 in neutral and alkaline conditions.
• There is a strong synergistic effect of Ag and Cu for the inactivation of MS2 at pH 7 and higher. No synergy was observed for PhiX 174.
• More work is needed to evaluate the efficiency of Cu and Ag in presence of organics to facilitate its further application as virucides for drinking water treatment.
• Although the concentrations used are higher than WHO recommendations, the study highlights the potential of Cu and Ag as possible virus disinfectants in (household) drinking water treatment.

## Declaration of competing interest

The authors declare that they have no known competing financial interests or personal relationships that could have appeared to influence the work reported in this paper.
