# Supplementary material for: Inactivation of RNA and DNA viruses in water by copper and silver ions and their synergistic effect
Source: Water Res X. 2020 Nov 5;9:100077. doi: 10.1016/j.wroa.2020.100077 (PMC7663217; doi:10.1016/j.wroa.2020.100077)
Supplement: Multimedia component 1 [file mmc1.docx]

**Supplementary material**

Inactivation of RNA and DNA viruses in water by copper and silver ions and their synergistic effect

Mona Y.M. Soliman ^a, *^, Gertjan Medema ^a,b^, Boris Estrada Bonilla ^c,d^, Stan J.J. Brouns ^c,d^, Doris van Halem ^a^

^a^ Department of Water Management, Delft University of Technology, Stevinweg 1, 2628 CN Delft, The Netherlands.  ^b^ KWR Watercycle Research Institute, P.O. Box 1072, 3430 BB, Nieuwegein, The Netherlands.
^c^ Department of Bionanoscience, Kavli Institute of Nanoscience, Delft University of Technology, Van der Maasweg 9, 2629 HZ Delft, The Netherlands.

^d^ Fagenbank, Van der Maasweg 9, 2629 HZ Delft, The Netherlands.

^*^ [M.Y.M.Soliman@tudelft.nl](mailto:M.Y.M.Soliman@tudelft.nl)

# Speciation of metal ions

**
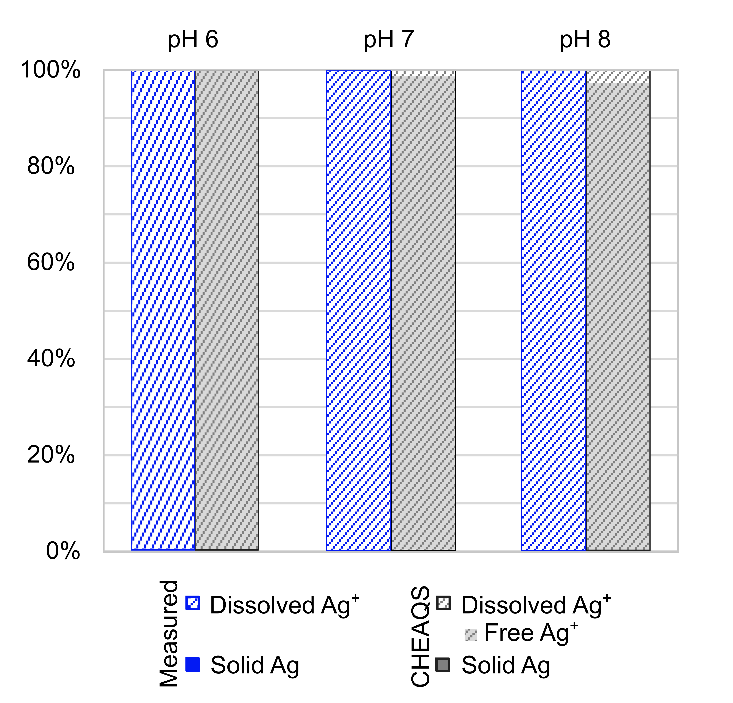
**

**Figure S1.** Speciation of Ag ions in solution expressed through experimental measurements of dissolved and solid Ag concentrations referred to as (measured) and simulated speciation using CHEAQS next chemical software. Ag measured concentrations after the passing 0.1 µm filter are expressed as dissolved, while the difference between dissolved and the unfiltered sample is considered solid complex. Values expressed as a percentage of the total Ag concentration which was used as the input for CHEAQS as well.

Table S1 Detailed speciation of the dissolved Cu as output of CHEAQS next modelling.

| Speciation (%) | pH 6 | pH 7 | pH 8 |
| --- | --- | --- | --- |
| Free Cu^2+^ | 16.3 % | 0.9 % | 0.2 % |
| CuHPO_4_ (aq) | 6.7 % | 3.3 % | 1.1 % |
| Cu(OH)^+^ | 0.3 % | 0.2 % | 0.2 % |
| CuSO_4_  (aq) | 0.2 % | 0.0 % | 0.0 % |
| CuH_2_(PO_4_)_2_ ^2-^ | 0.1 % | 0.7 % | 0.4 % |
| Total dissolved Cu (%) | 23.6 % | 5.2 % | 1.9 % |

# Antiviral activity of Cu and Ag combined

**
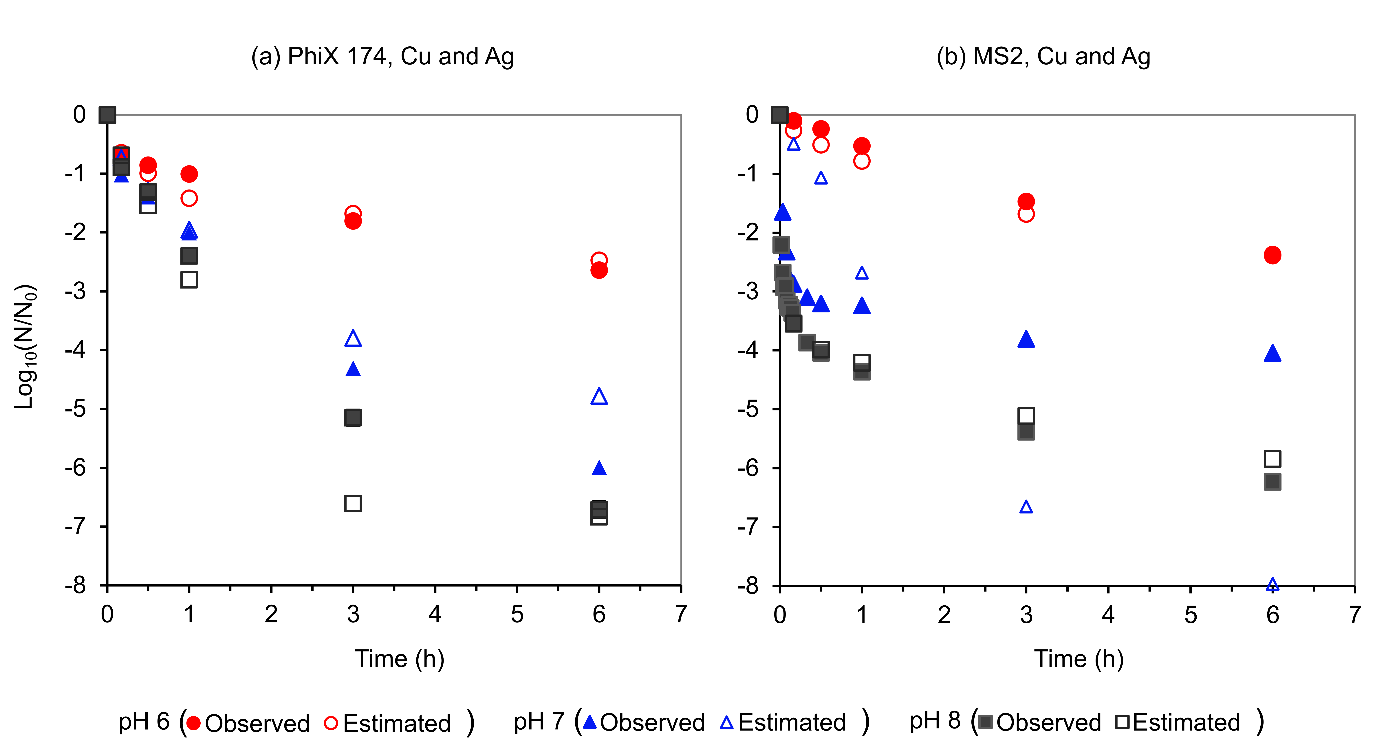
Figure S2.** Six hours inactivation of (a) PhiX 174 and (b) MS2 by Cu and Ag ions combined (Observed) and the mathematical sum of LRVs obtained through individual treatment (Estimated).


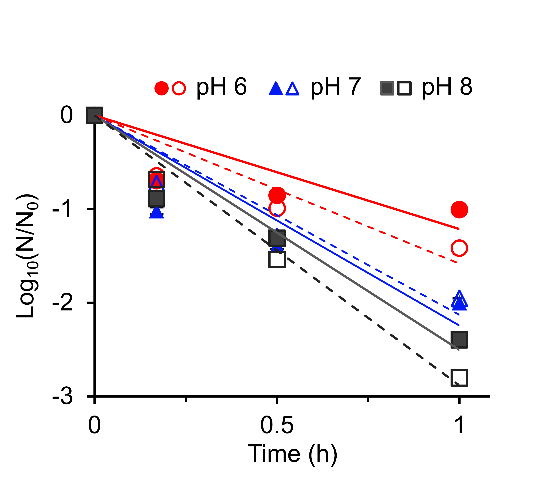


**Figure S3.** First hour inactivation kinetics of PhiX 174 by Cu and Ag combined as observed (closed marker) and the mathematical sum of LRVs obtained through individual treatment as estimated (open marker).

# Morphological changes of MS2 and PhiX 174

The protocol for imaging MS2 and PhiX 174 samples is described in the main manuscript (Section 2.6). To ensure that the sample represents the inactivation experiment, samples prepared for imaging were also neutralized, diluted and quantified. Initially 300 µl of MS2 or PhiX 174 were divided into 3 parts (each of 100 µl). One aliquot was treated as control, another treated with Cu and the last with Ag. From each aliquot, a 50 µl sample was neutralized and diluted in PBS. A 10 µl of each dilution was deposited on agar plates containing host bacterium (Jebri et al., 2017). Lysis zones were evaluated for log inactivation values (LRV) compared to the control.

Imaged MS2 samples had viable phage concentrations lower than the control by ~ 1.5 logs in case of Cu and ~ 5 logs in case of Ag. PhiX samples had phage concentrations lower than control by ~0.2 logs in case of Cu and by ~4 logs in case of Ag. Images of control samples are referred to as intact.


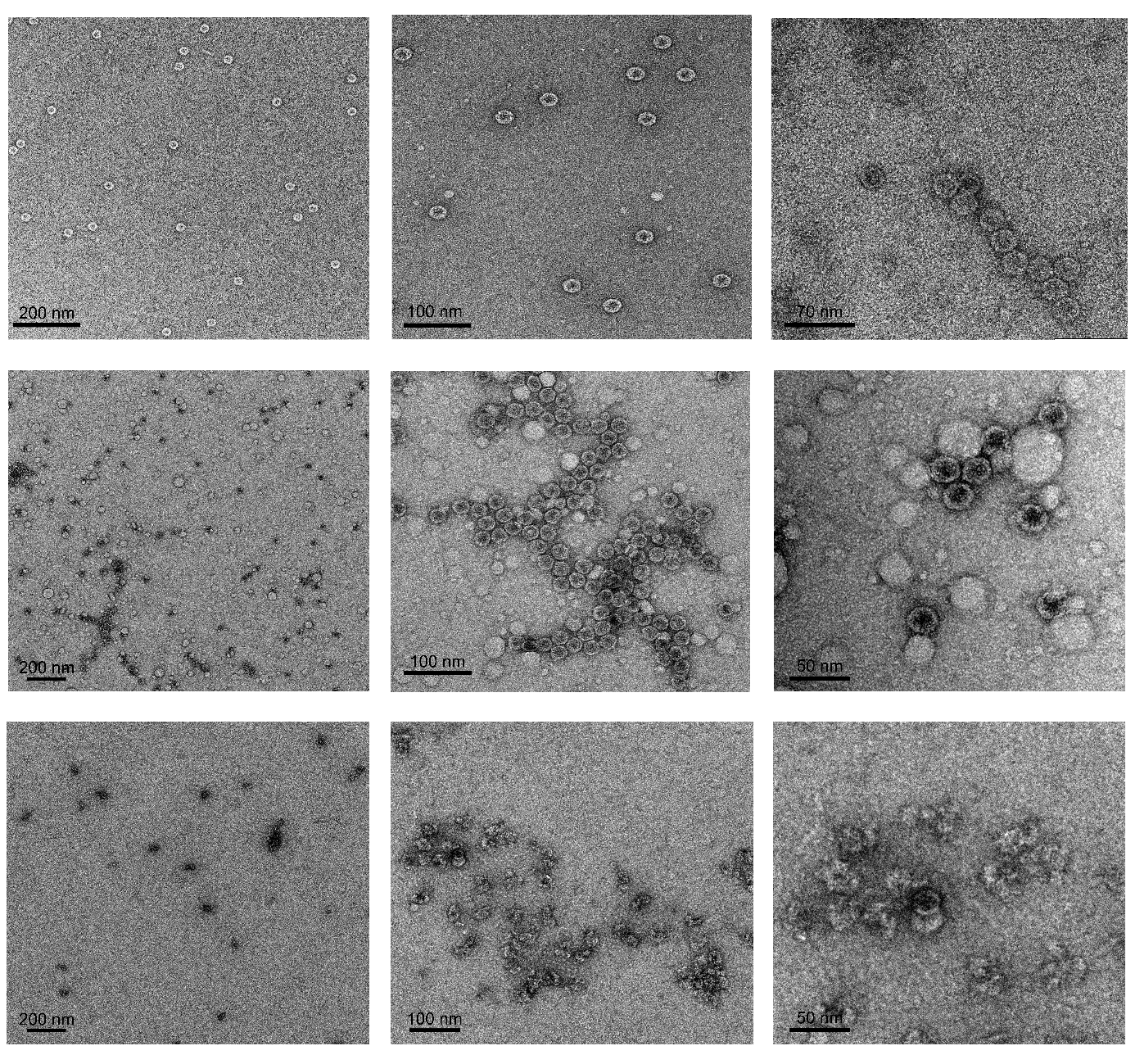


**Figure S4.** Additional TEM images of MS2 showing intact particles (top row), Cu treated particles (middle row) and Ag treated particles (bottom row).


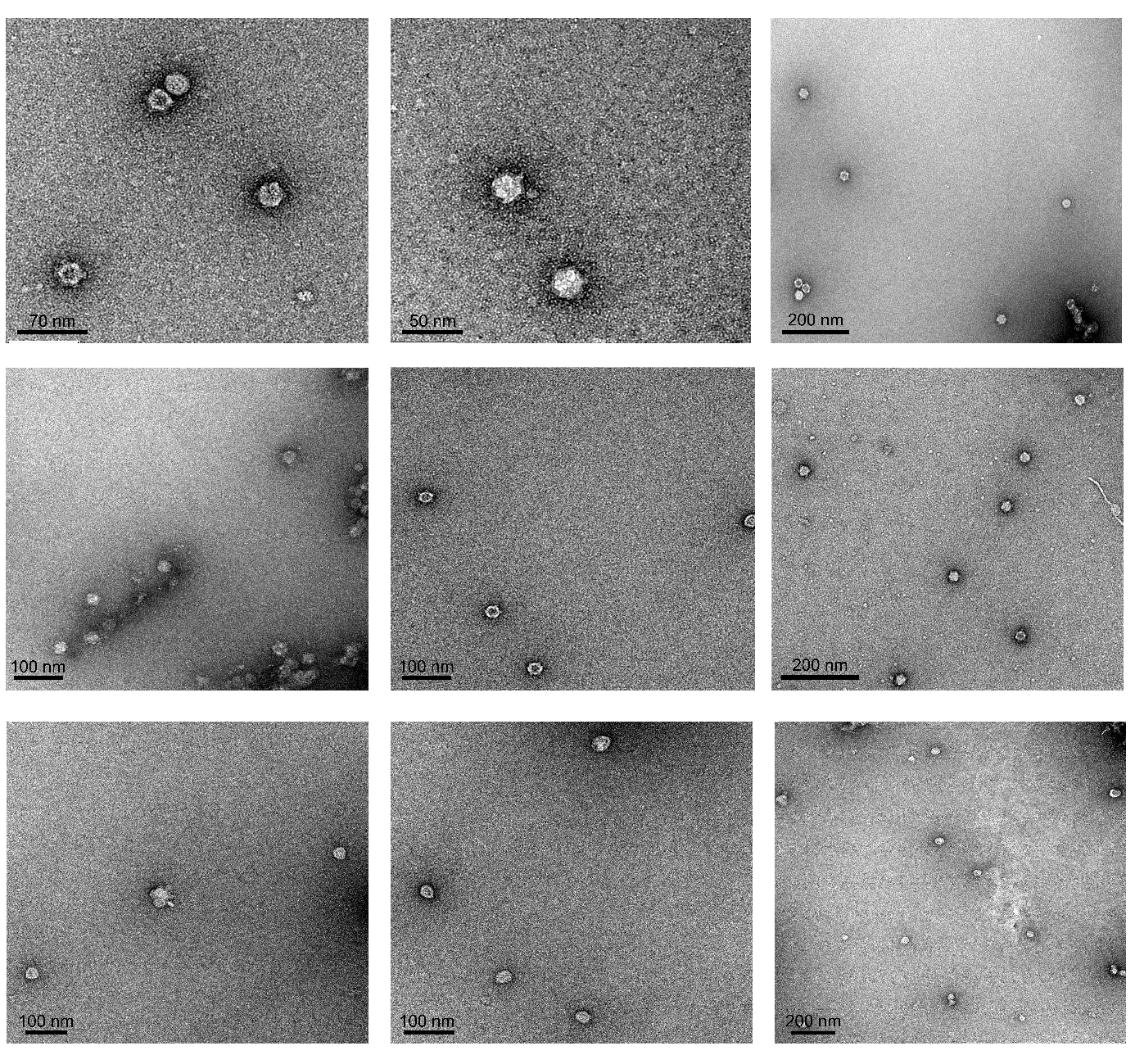


**Figure S5.** Additional TEM images of PhiX 174 showing intact particles (top row), Cu treated particles (middle row) and Ag treated particles (bottom row).

# Modelling of amino acids pKa values for MS2 and PhiX 174

Predicting pKa values of amino acids in the protein structures of MS2 and PhiX 174 was carried out using PROPKA (version 3.0) and PARSE forcefield (Dolinsky et al., 2004). Protonation state was assigned at pH 6, 7 and 8. Since negligible difference in pKa values was observed using different pH entries, the output is provided at pH 7 (Table S2, S3 and S4). The protein structures from the Protein Data Bank (PBD) 2MS2 and 1RB8 were provided as input for MS2 and PhiX 174 respectively.

**Table S2.** pKa values of MS2 coat protein predicted at pH 7 using PROPKA (version 3.0)

| Aminoacid | pKa | Aminoacid | pKa |
| --- | --- | --- | --- |
| ASP 11 | 4.0 | LYS 66 | 10.4 |
| ASP 17 | 3.8 | GLU 76 | 4.7 |
| GLU 31 | 4.7 | ARG 83 | 11.9 |
| ARG 38 | 11.2 | TYR 85 | 10.4 |
| TYR 42 | 10.6 | GLU 89 | 3.9 |
| LYS 43 | 10.1 | ASP 100 | 3.0 |
| CYS 46 | 10.4 | CYS 101 | 9.9 |
| ARG 49 | 12.3 | GLU 102 | 4.7 |
| ARG 56 | 12.8 | LYS 106 | 10.4 |
| LYS 57 | 10.7 | LYS 113 | 10.4 |
| TYR 58 | 9.9 | ASP 114 | 3.9 |
| LYS 61 | 10.4 | TYR 129 | 10.2 |
| GLU 63 | 4.3 |  |  |

**Table S3.** pKa values of PhiX 174 spike protein (G) predicted at pH 7 using PROPKA (version 3.0)

| Aminoacid | pKa | Aminoacid | pKa | Aminoacid | pKa | Aminoacid | pKa |
| --- | --- | --- | --- | --- | --- | --- | --- |
| ARG 101 | 11.9 | ASP 61 | 4.0 | GLU 116 | 4.7 | LYS 187 | 10.3 |
| ARG 135 | 12.2 | ASP 71 | 2.9 | GLU 118 | 2.4 | LYS 69 | 10.8 |
| ARG 145 | 16.2 | ASP 76 | 4.7 | GLU 123 | 4.6 | TYR 120 | 11.7 |
| ARG 166 | 12.4 | CYS 132 | 7.7 | GLU 179 | 4.7 | TYR 180 | 9.4 |
| ARG 17 | 12.1 | CYS 137 | 10.2 | GLU 44 | 10.8 | TYR 2 | 10.0 |
| ARG 45 | 12.1 | CYS 157 | 16.9 | HIS 119 | 5.8 | TYR 51 | 9.8 |
| ASP 10 | 4.0 | CYS 79 | 11.0 | HIS 143 | 5.8 | TYR 94 | 10.1 |
| ASP 136 | 3.1 | GLU 103 | 2.8 | HIS 178 | 6.2 | ARG 10 | 12.4 |
| ASP 141 | 2.6 | GLU 107 | 4.3 | LYS 161 | 10.4 | ARG 13 | 12.5 |
| LYS 187 | 10.3 | TYR 120 | 11.7 | TYR 2 | 10.0 |  |  |
| LYS 69 | 10.8 | TYR 180 | 9.4 | TYR 51 | 9.8 |  |  |

**Table S4.** pKa values of PhiX 174 capsid protein (F) predicted at pH 7 using PROPKA (version 3.0)

| Aminoacid | pKa | Aminoacid | pKa | Aminoacid | pKa | Aminoacid | pKa |
| --- | --- | --- | --- | --- | --- | --- | --- |
| ARG 10 | 12.1 | ARG 56 | 12.2 | ASP 382 | 3.8 | GLU 280 | 4.7 |
| ARG 102 | 12.4 | ARG 57 | 12.2 | ASP 394 | 6.0 | GLU 299 | 7.8 |
| ARG 119 | 12.3 | ARG 75 | 13.9 | ASP 396 | 6.7 | GLU 328 | 4.4 |
| ARG 137 | 11.3 | ARG 87 | 12.6 | ASP 40 | 4.8 | GLU 348 | 5.2 |
| ARG 144 | 13.5 | ASP 105 | 3.6 | ASP 426 | 4.0 | GLU 386 | 3.9 |
| ARG 158 | 13.2 | ASP 14 | 3.3 | ASP 45 | 4.3 | GLU 43 | 4.8 |
| ARG 162 | 12.3 | ASP 155 | 4.0 | ASP 62 | 6.3 | HIS 106 | 5.5 |
| ARG 209 | 12.3 | ASP 156 | 3.6 | ASP 66 | 4.0 | HIS 125 | 4.5 |
| ARG 215 | 9.3 | ASP 192 | 3.4 | ASP 80 | 4.0 | HIS 165 | 3.3 |
| ARG 217 | 11.8 | ASP 21 | 4.0 | ASP 88 | 4.0 | HIS 17 | 6.1 |
| ARG 234 | 12.9 | ASP 218 | 5.4 | CYS 100 | 10.8 | HIS 204 | 6.3 |
| ARG 264 | 13.3 | ASP 230 | 2.7 | CYS 163 | 12.8 | HIS 240 | 5.9 |
| ARG 27 | 11.0 | ASP 232 | 3.5 | CYS 164 | 13.4 | HIS 271 | 3.6 |
| ARG 275 | 12.9 | ASP 242 | 4.9 | CYS 22 | 11.3 | HIS 281 | 5.5 |
| ARG 291 | 15.4 | ASP 249 | 4.3 | CYS 398 | 12.2 | HIS 300 | 5.4 |
| ARG 327 | 13.9 | ASP 251 | 4.0 | GLU 11 | 4.7 | HIS 301 | 5.5 |
| ARG 332 | 12.4 | ASP 254 | 2.5 | GLU 143 | 4.6 | HIS 355 | 6.8 |
| ARG 336 | 12.4 | ASP 313 | 2.3 | GLU 146 | 4.3 | HIS 364 | 6.4 |
| ARG 339 | 12.3 | ASP 317 | 3.1 | GLU 154 | 4.8 | HIS 367 | 6.1 |
| ARG 353 | 11.9 | ASP 333 | 3.3 | GLU 177 | 4.9 | HIS 392 | 2.1 |
| ARG 391 | 12.6 | ASP 337 | 4.3 | GLU 182 | 4.0 | HIS 420 | 5.9 |
| ARG 412 | 12.4 | ASP 357 | 5.0 | GLU 183 | 4.7 | HIS 74 | 3.7 |
| ARG 419 | 12.0 | ASP 365 | 3.2 | GLU 187 | 4.4 | HIS 76 | 5.4 |
| ARG 425 | 12.3 | ASP 373 | 7.0 | GLU 206 | 4.9 | LYS 122 | 9.9 |
| ARG 51 | 11.7 | ASP 374 | 4.8 | GLU 208 | 5.6 | LYS 167 | 8.5 |
| LYS 179 | 11.4 | LYS 64 | 10.6 | TYR 211 | 11.4 | TYR 358 | 10.3 |
| LYS 270 | 10.5 | TYR 103 | 10.1 | TYR 216 | 10.3 | TYR 363 | 10.2 |
| LYS 29 | 10.6 | TYR 109 | 15.0 | TYR 229 | 11.7 | TYR 395 | 14.1 |
| LYS 306 | 11.2 | TYR 128 | 15.4 | TYR 248 | 9.3 | TYR 413 | 13.9 |
| LYS 343 | 10.6 | TYR 132 | 14.1 | TYR 302 | 10.8 | TYR 418 | 10.0 |
| LYS 345 | 10.0 | TYR 135 | 11.7 | TYR 311 | 11.4 | TYR 71 | 13.6 |
| LYS 362 | 10.5 | TYR 159 | 12.1 | TYR 331 | 14.0 | TYR 78 | 11.8 |
| LYS 409 | 7.7 | TYR 200 | 10.1 | TYR 352 | 10.5 |  |  |
